# Efficacy and safety of pembrolizumab in patients with advanced endometrial cancer: a systematic review and meta-analysis

**DOI:** 10.3389/fonc.2024.1511301

**Published:** 2025-02-04

**Authors:** Biqiong Pan, Xiaojun Lai, Jiefang Lu, Xiaoyan Bao, Zengke Fan, Jie Sun

**Affiliations:** ^1^ Department of Gynecology and Obstetrics, Lishui People’s Hospital, Lishui, Zhejiang, China; ^2^ Department of Anorectal Surgery, Lishui People’s Hospital, Lishui, Zhejiang, China

**Keywords:** pembrolizumab, monotherapy, endometrial cancer, meta-analysis, efficacy and safety

## Abstract

**Objective:**

This meta-analysis evaluated pembrolizumab monotherapy and combination therapy’s efficacy and safety in recurrent or advanced endometrial cancer (EC).

**Methods:**

We utilized PubMed, Embase, Cochrane Library, and Web of Science databases to identify clinical trials that were used to search literature from July 2013 to July 2023 to evaluate the efficacy and safety of pembrolizumab in patients with advanced EC. Eight studies with 2,742 patients were included. Outcomes were progression-free survival (PFS), overall survival (OS), objective response rate (ORR), complete remission (CR), and adverse events (AEs); a subgroup analysis was carried out based on combination treatment regimens. Quality assessment of the included studies was conducted using the Cochrane Risk of Bias Tool, the Newcastle–Ottawa Scale (NOS), and the Joanna Briggs Institute (JBI) critical appraisal checklist.

**Results:**

Pembrolizumab reduced progression risk [hazard ratio (HR): 0.53; 95% confidence interval (CI): 0.44, 0.63; *p* < 0.00001] and death risk when combined with lenvatinib (HR: 0.67; 95% CI: 0.59, 0.76; *p* < 0.00001). Pembrolizumab monotherapy and lenvatinib combination achieved higher ORR (OR: 3.61; 95% CI: 2.12, 6.13; *p* < 0.00001) and CR rates (OR: 2.7; 95% CI: 1.59, 4.57; *p* < 0.05) than controls. Single-arm studies: 8% CR and 4% PR in pembrolizumab-treated patients. Pooled AE incidence: 86%, with 43% grade 3/4. Two randomized controlled trials (RCTs) found that the pembrolizumab group had a higher incidence of grade 3 or 4 AEs compared to the control group (OR: 2.23; 95% CI: 1.23, 4.04; *p* = 0.008).

**Conclusion:**

Pembrolizumab monotherapy or combination significantly improves survival in recurrent or advanced EC and has manageable toxicity albeit with a relatively high incidence of treatment-related AEs.

## Introduction

As the second most common gynecological malignancy worldwide, endometrial cancer (EC) has a rising incidence globally. Approximately 66,200 new cases and 13,030 deaths among women will occur in 2023. EC may become the third most prevalent and fourth leading cause of cancer-related mortality among women by 2040 (1, 2). The survival time of patients is closely related to the stage of the disease. A large number of patients diagnosed with stage I or II EC have a favorable prognosis, with a 5-year survival rate of approximately 90%. However, roughly 10%–13% of patients with recurrent or stage III–IV advanced EC face treatment challenges and have a poor overall prognosis. The 5-year survival rate is below 50% for patients with lymph node metastasis and lower than 20% for those with peritoneal or distant metastasis (3–5). Uterine corpus cancer mortality rates rise by approximately 1% annually. The prognosis for women with advanced EC is poor due to the lack of major treatment advances (6).

Until now, treatment for advanced EC has included conventional platinum-based chemotherapy or hormone therapy. The oncology revolution represented by the advent of immune checkpoint inhibitors (ICIs) has also led to significant advances in the management of recurrent and metastatic EC (7). Malignant tumors possess the ability to evade immune surveillance, a mechanism stemming from either the absence of tumor cell antigen expression or the establishment of an immunotolerant environment (8). Melanoma, non-small cell lung cancer, and digestive system tumors are treated by targeting the programmed death receptor 1/programmed death ligand 1 (PD-1/PD-L1) signaling pathway, which is also the target of ICIs (9–12).

Selecting therapies beyond first-line drugs for EC is complicated by the diverse histologic, molecular, and clinical features of the disease. The Cancer Genome Atlas (TCGA) classifies EC into specific subgroups based on genomic features, which have been demonstrated as reliable prognostic biomarkers (13). These subtypes include POLE mutant, Microsatellite Instability High (MSI-H) or deficient in Mismatch Repair System (dMMR), TP53 mutant, and no specific molecular profile (14). The overlap between dMMR and MSI-H tumor status is high (approximately 90%–95%); thus, the two states are considered interchangeable (15). Studies have demonstrated the established efficacy of ICI monotherapy, such as pembrolizumab and dostarlimab-gxly (16), as second-line and beyond treatment for EC patients with MSI-H and dMMR (17). These agents reactivate T cell-mediated anti-tumor immunity by inhibiting the PD-1/PD-L1 immune checkpoint pathway. Several published clinical studies have investigated pembrolizumab for EC; however, their results demonstrate significant variations. This review evaluated the efficacy and safety of pembrolizumab for EC by analyzing data from relevant clinical trials.

## Materials and methods

### Literature search

According to the principles in the Preferred Reporting Items for Systematic Reviews and Meta-Analyses (PRISMA) guidelines, our meta-analysis was conducted (18). Meanwhile, this study was also registered in the PROSPERO database (registration number: CRD42024522789). Four databases including PubMed, Embase, Cochrane Library, and Web of Science were used to search literature from July 2013 to July 2023. MeSH terms were employed to search the databases and search with the following keywords: (“pembrolizumab” OR “SCH-900475” OR “lambrolizumab” OR “MK-3475” OR “Keytruda” AND (“endometrial cancer” OR “endometrial carcinoma” OR “endometrium cancer” OR “endometrial neoplasms”).

### Identification of eligible studies

The selected studies’ inclusion criteria were as follows: (1) studies involving adult participants diagnosed with advanced-stage endometrial malignancies; (2) trials assessing the use of pembrolizumab, either as a standalone treatment or in combination with other therapeutic agents; and (3) studies reporting relevant clinical outcomes, such as the specific drug administered, survival outcomes [progression-free survival (PFS) and overall survival (OS)], tumor response rates [objective response rate (ORR) and complete remission (CR)], and the incidence of severe adverse events (AEs) (grade 3, 4, or 5 toxicities). The following types of publications were excluded from the analysis: review articles, commentaries, opinion pieces, individual case reports, abstracts from scientific meetings, studies focusing on pediatric populations, unpublished manuscripts, and research articles not written in the English language.

### Data extraction

Data such as drug name, population, PFS, OS, ORR, CR, grade 3 or 4 AE, and grade 5 AE were extracted from included studies. A preliminary validity and relevance analysis was conducted for the included articles. Initially, relevant studies were identified and unrelated studies were excluded according to the title and abstract. Subsequently, a full-text analysis of the selected studies that met the eligibility criteria was performed, followed by data extraction. Furthermore, a pair of reviewers independently examined the bibliographies of all included studies to identify additional relevant articles. In case of discrepancies during the literature search process, the reviewers engaged in discussions to reach a mutual agreement.

### Quality assessment

Randomized controlled trials’ (RCTs’) risk bias was evaluated using the Cochrane Risk of Bias Tool (19) (**Supplementary Figure S1**). The methodological quality of the selected studies was assessed using the Newcastle–Ottawa Scale (NOS) (20), with scores ranging from seven to nine indicating high-quality research (21) (**Supplementary Table S1**). For single-arm studies, the Joanna Briggs Institute (JBI) critical appraisal checklist was employed to determine the quality (**Supplementary Table S2**). In RCTs, the risk of bias was examined across various domains, including participant selection, intervention performance, outcome detection, participant attrition, reporting, and other potential sources of bias. Each domain was categorized as having a “low risk,” “high risk,” or “unclear risk” of bias. Regarding retrospective studies, NOS scores of 0–4, 5–7, and 8–9 were considered indicative of low, moderate, and high quality, respectively, corresponding to high, moderate, and low risks of bias. Two independent reviewers conducted the quality assessment and determined the level of evidence for each included study, with any disagreements resolved through discussion until a consensus was reached.

### Statistical analysis

Data extraction and forest plot creation for RCTs and cohort studies were conducted using Review Manager 5.4 (Cochrane Collaboration, Oxford, UK), while single-arm studies were analyzed using STATA (version 17, College Station, TX, USA). Continuous and dichotomous variables were assessed using hazard ratios (HRs) and ORs, respectively, with 95% CIs reported for all effect estimates. Heterogeneity among the included studies was determined using Cochrane’s *Q* statistic and *I*-squared (*I*²) statistics (22). Significant heterogeneity was defined as a Cochrane *Q*-test *p*-value < 0.05 or *I*² > 50%, in which case a random-effects model was employed to calculate the pooled HR or OR; otherwise, a fixed-effects model was used. To assess the impact of significant heterogeneity on the combined effect estimates, one-way sensitivity analyses were performed. Egger’s regression tests (23) in STATA (version 17, College Station, TX, USA) was used to evaluate the publication bias, with *p* < 0.05 implying statistically significant bias. Considering that the efficacy of pembrolizumab may be affected by combination or adjuvant treatment regimens (17, 24), a subgroup analysis based on combination treatment regimens was performed in this study to explore whether there is a difference in the efficacy of pembrolizumab under different combination treatment regimens and whether it leads to heterogeneity.

## Results

### Literature search and study characteristics

**Figure 1:**
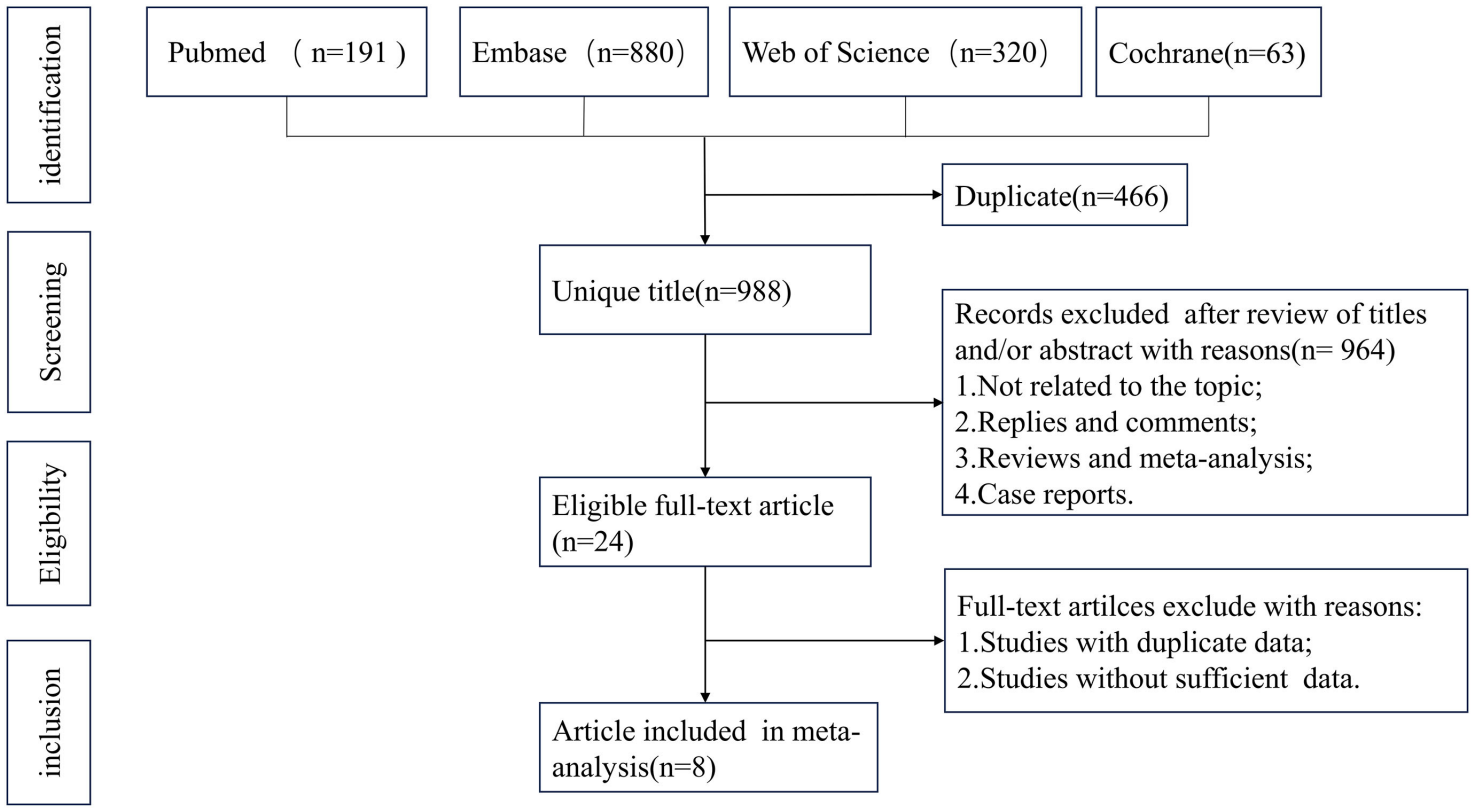
Flowchart of the systematic search and selection process.

The system retrieval and selection process is shown in **Figure 1**. There were 1,454 articles retrieved from PubMed (*n* = 191), Embase (*n* = 880), Web of Science (*n* = 320), and Cochrane (*n* = 63). After removing duplicates, 988 articles remained. Finally, 283 studies were excluded, leaving 8 full-text articles involving 2,742 patients for meta-analysis. Two were prospective randomized studies (17, 24), five were single-arm studies (25–29), and one was a retrospective cohort study (15). **Supplementary Table S3** shows the characteristics. In the two prospective randomized studies, the mismatch repair proficient (pMMR) population was defined as Group a, and the overall population was defined as Group b in Makker et al.’s study (24). In Eskander et al.’s study (17), the pMMR population was defined as Group a, and the dMMR population was defined as Group b.

### Efficacy of pembrolizumab in EC based on PFS

**Figure 2:**
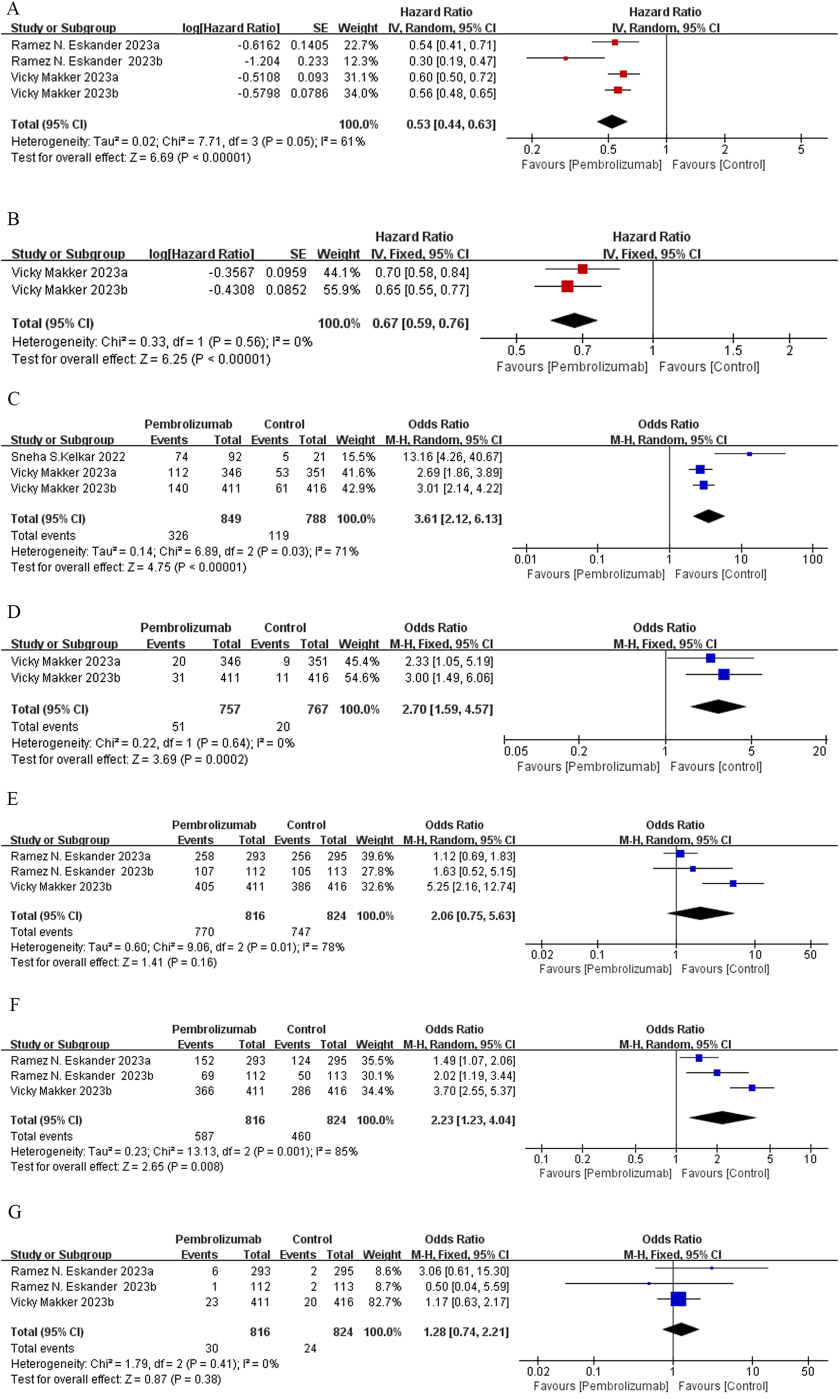
Forest plots of RCT outcomes: **(A)** PFS, **(B)** OS, **(C)** ORR, **(D)** CR, **(E)** AE, **(F)** grade 3 or 4 AE, and **(G)** grade 5 AE.

**Figure 3:**
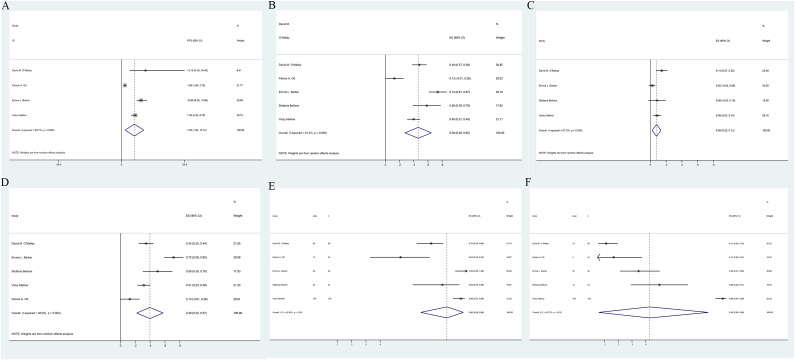
Forest plots of single-arm study outcomes: **(A)** PFS, **(B)** ORR, **(C)** CR, **(D)** PR, and **(E)** AE. **(F)** grade 3 or 4 AE.

Meta-analysis of two RCTs (17, 24) demonstrated that pembrolizumab decreased by 47% the advanced EC patients’ disease progression risk compared to the control group [HR: 0.53; 95% confidence interval (CI): 0.44, 0.63; *p* < 0.00001], despite considerable heterogeneity among the studies (*I*² = 61%, *p* = 0.05) (**Figure 2A**). Subgroup analysis based on treatment regimens showed that pembrolizumab combined with chemotherapy had a lower progression rate than pembrolizumab plus lenvatinib [(HR: 0.41; 95% CI: 0.23, 0.74) vs. (HR: 0.58; 95% CI: 0.51, 0.65)]. Differences in treatment approaches could explain the observed heterogeneity (**Supplementary Figure S2A**). Funnel plot implied and Egger’s test indicated low risk of publication bias (*p* = 0.147) (**Supplementary Figure S3A**). Data pooled from five single-arm studies (25–29) estimated a median PFS of 7.03 months (95% CI: 1.94, 12.12), with heterogeneity among the studies (*I*² = 95.7%, *p* = 0.000) (**Figure 3A**).

### Efficacy of pembrolizumab in EC based on OS

One RCT study (24) assessing OS divided 1,524 patients with advanced EC into two groups based on their MMR status: mismatch pMMR (Group a) and the total population (Group b). The trial demonstrated that pembrolizumab combined with lenvatinib significantly reduced the risk of mortality in EC patients compared to conventional chemotherapy (HR: 0.67; 95% CI: 0.59, 0.76; *p* < 0.00001) (**Figure 2B**), with no heterogeneity observed among the subgroups (*I*² = 0%, *p* = 0.56). The funnel plot indicated no publication bias (**Supplementary Figure S3B**).

### Efficacy of pembrolizumab in EC based on ORR, CR, and PR

Meta-analysis of two clinical studies (15, 24) involving 1,637 advanced EC patients demonstrated that pembrolizumab, either as monotherapy or combined with lenvatinib, achieved significantly higher ORR compared to control groups (OR: 3.61; 95% CI: 2.12, 6.13; *p* < 0.00001) (**Figure 2C**). The funnel plot and Egger’s test were not statistically significant (*p* = 0.161) (**Supplementary Figure S3C**). Data pooled from five single-arm trials (25–29) estimated an overall ORR of 46% (95% CI: 0.28, 0.65), despite heterogeneity among the studies (*I*² = 91.4%, *p* = 0.000) (**Figure 3B**). Patients receiving pembrolizumab, either alone or combined with lenvatinib, had a significantly higher pooled CR rate compared to control groups (OR: 2.7; 95% CI: 1.59, 4.57; *p* < 0.05), with no heterogeneity observed (*I*² = 0%, *p* = 0.64) (**Figure 2D**). The included single-arm studies indicated that advanced EC patients treated with pembrolizumab had a CR rate of 8% (95% CI: 0.02, 0.12; *p* < 0.05) and a PR rate of 4% (95% CI: 0.22, 0.57; *p* < 0.05) (**Figures 3C, D**).

### Safety of pembrolizumab in EC

Two randomized controlled trials, one comparing pembrolizumab plus chemotherapy versus chemotherapy with placebo (17) and the other comparing pembrolizumab and lenvatinib versus chemotherapy (24), were meta-analyzed. No statistically significant differences were found in the incidence of treatment-related AEs (OR: 2.06; 95% CI: 0.75, 5.63; *p* = 0.16), despite substantial heterogeneity between the studies (*I*² = 78%, *p* = 0.01) (**Figure 2E**). The incidence of grade ≥5 AEs was not significantly different between the groups (OR: 1.28; 95% CI: 0.74, 2.21; *p* = 0.38) (**Figure 2G**). However, patients receiving pembrolizumab experienced grade 3 or 4 AEs more frequently compared to those in the control group (OR: 2.23; 95% CI: 1.23, 4.04; *p* = 0.008), with considerable heterogeneity (*I*² = 85%, *p* = 0.001) (**Figure 2F**). Subgroup analysis based on the administration method suggested that differences in dosing regimens could explain the observed heterogeneity (**Supplementary Figure S2B**). The funnel plot’s visual assessment and Egger’s test results did not indicate any selection bias (*p* > 0.05). Data pooled from five single-arm studies evaluating pembrolizumab (25–29) estimated an AE incidence of 86% (95% CI: 0.68, 0.98), with substantial heterogeneity among the studies (*I*² = 92%, *p* = 0.00) (**Figure 3E**). The estimated incidence of grade 3 or 4 treatment-related AEs was 43% (95% CI: 0.06, 0.86), with significant heterogeneity (*I*² = 98.27%, *p* = 0.00) (**Figure 3F**).

### Sensitivity analysis

**Figure 4:**
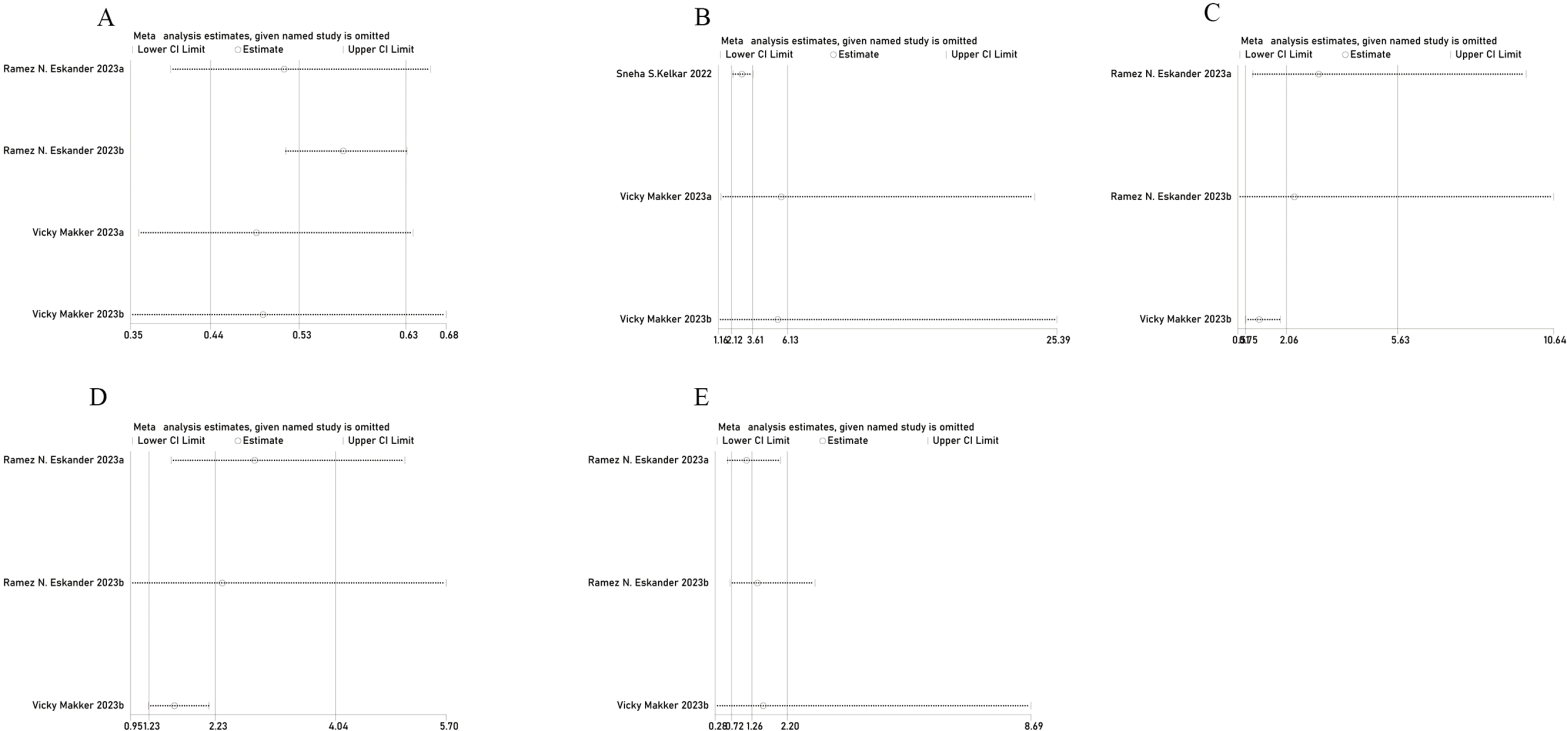
Sensitivity analysis of **(A)** PFS, **(B)** ORR, **(C)** AE, **(D)** grade 3 or 4 AE, and **(E)** grade 5 AE.

The sensitivity analysis demonstrated that after excluding any individual study in PFS, ORR, AE, or grade 3 or 4 AE, the new combined HR or OR remained unchanged, indicating stable results (**Figure 4**). In the grade 5 AE analysis, excluding the Eskander et al. Group b data (17) changed the results from statistically significant to non-significant, suggesting unstable results. Therefore, it cannot be concluded that the combination of pembrolizumab does not increase the incidence of grade 5 AE in patients with EC. However, excluding Group b data reported by Eskander et al. in 2023 (17) reduced the heterogeneity of PFS from 71% to 0%. Similarly, excluding Kelkar et al. (15) decreased ORR heterogeneity from 71% to 0%. Furthermore, excluding data reported by Makker et al. (28) reduced the heterogeneity of AE from 78% to 0% and grade 3 AE heterogeneity from 85% to 0%, indicating that the excluded studies accounted for most of the heterogeneity.

## Discussion

Immunotherapy has emerged as a novel approach for treating tumors over the past decade, with a current focus on PD-1/PD-L1 inhibitors. The PD-1 signaling pathway inhibits T-cell activation, regulating newborn T cells and preventing immune responses to normal tissues. During cancer progression, the upregulation of PD-1 ligands (PD-L1 and PD-L2) on tumor or immune cells in the tumor microenvironment activates this immunosuppressive pathway, enabling tumors to evade anti-tumor immune surveillance (30). Targeted therapy against PD-1 removes this blockade, and the immune system recognizes and destroys tumor cells (31). Based on successful clinical trials, the Food and Drug Administration (FDA) has approved several α-PD-1 antibodies, including nivolumab and pembrolizumab, for the treatment of various cancer types, such as lymphoma, kidney cancer, and bladder cancer (32).

OS is a crucial outcome in cancer patient treatment and the ultimate endpoint of phase III clinical trials. In recent years, with the increasing number of new cancer drugs approved, PFS has become an alternative endpoint to OS in many cancer clinical trials and is used as the primary endpoint to inform regulatory agencies and clinical practice (33). This meta-analysis revealed that the PFS of recurrent advanced EC patients treated with pembrolizumab was 7.03 months, based on data from five single-arm studies (25–29). For monotherapy, high ORRs are associated with regulatory approval, and an ORR exceeding 30% is considered a statistically significant and appropriate endpoint for single-arm trials demonstrating breakthrough activity of monotherapy against cancer (34). The pooled ORR proportion in the five included single-arm studies was 46.0%, significantly surpassing the 30% threshold. The results of the included case–control and cohort studies showed that the pembrolizumab group had a 3.61-fold higher OR for achieving ORR compared to the chemotherapy group. Combined therapy approaches have been actively explored in EC clinical trials in addition to monotherapy immunotherapy.

Pembrolizumab combined with chemotherapy has been shown to significantly improve PFS and OS in various solid tumor patients (35, 36). These improvements are thought to result from increased tumor antigen diversity due to impaired point mutation repair in tumor cells and the potential immunogenic effects of cytotoxic chemotherapy, including myeloid-derived suppressor cell inhibition, enhanced antigen cross-presentation following immunogenic cell death, increased dendritic cell activity via STAT 6 pathway inhibition, and elevated proportions of cytotoxic lymphocytes and regulatory T cells (27). The data of Eskander et al. (17) were divided into two cohorts, namely, dMMR and pMMR; these two cohorts were separated and did not affect each other, and even if the data in one group do not reach a positive result, it does not affect the result analysis of the other group. The final results demonstrated significantly prolonged PFS in dMMR and pMMR EC patients by adding pembrolizumab to standard chemotherapy followed by pembrolizumab maintenance therapy, with a 70% reduced risk of disease progression or death in the dMMR cohort and 46% in the pMMR cohort, suggesting that incorporating immunotherapy in first-line treatment for advanced or recurrent EC improves oncological outcomes, regardless of MMR status or histological findings. Lenvatinib is a multi-targeted tyrosine kinase inhibitor, and the combination of pembrolizumab and lenvatinib is designed to enhance anti-tumor immune responses and exert anti-angiogenic effects (37). Makker et al. demonstrated significantly prolonged PFS and OS with pembrolizumab and lenvatinib compared to chemotherapy in the pMMR subgroup and the overall population of advanced EC patients progressing after prior systemic platinum-based therapy; the HR for progression or death was 0.60 (95% CI, 0.50 to 0.72; *p* < 0.001) and 0.56 (95% CI, 0.47 to 0.66; *p* < 0.001), respectively (24), suggesting that pembrolizumab immunotherapy is an effective treatment modality for recurrent advanced EC, exhibiting superior therapeutic outcomes. There was high heterogeneity in our study. Through sensitivity analysis, we found that the heterogeneity in PFS and AE analysis may have come from different treatment regimens. Makker’s treatment regimens was lenvatinib and pembrolizumab, but Eskander’s was pembrolizumab and chemotherapy. ORR heterogeneity may due to different research methods, Kelkar’s research was retrospective, but others were RCTs.

As a monoclonal antibody targeting PD-1, pembrolizumab can inhibit the interaction between PD-1 and its ligands, PD-L1 and PD-L2. The approved treatment options have changed the outlook for patients with recurrent or advanced EC in recent years. Prior to 2023, immunotherapy focused on second-line and posterior treatment of advanced metastatic or recurrent EC. Based on the outcome of Keynote-158 (25), the FDA granted successive approval for pembrolizumab monotherapy for ECs with MSI-H/dMMR that had progressed after frontline systemic therapy and was not amenable to curative surgery or radiation therapy in March 2022. On the basis of Keynote-146 (24), pembrolizumab combined with lenvatinib was also approved by the FDA in September 2019 for patients with non-MSI-H/pMMR tumors (38, 39). In June 17 of this year, the FDA approved pembrolizumab in combination with chemotherapy followed by pembrolizumab monotherapy for first-line treatment of advanced or recurrent EC due to the results of GY018 (40).

Five single-arm studies (25–29) revealed a pooled incidence of 86% for any AE and 43% for grade 3 or 4 treatment-related AEs. The two included prospective randomized studies (17, 24) indicated that the treatment of pembrolizumab did not increase the risk of AEs in general (OR: 2.06; 95% CI: 0.75, 5.63; *p* = 0.16). However, the pembrolizumab group had a higher incidence of grade 3 or 4 AEs compared to the control group (OR: 2.23; 95% CI: 1.23, 4.04; *p* = 0.008). The most common treatment-related AEs were hypertension, elevated lipase, fatigue, and diarrhea. Serious adverse reactions may require dose reduction, treatment interruption, or discontinuation of the trial drug. Substantial heterogeneity was observed, which might be attributed to different administration methods based on further investigation. The grade 5 AEs were not significantly different between the groups (OR: 1.28; 95% CI: 0.74, 2.21; *p* = 0.38); it seems to mean that the safety of pembrolizumab is manageable. It is worth mentioning that sensitivity analysis revealed instability in the incidence of grade 5 AEs, suggesting that the combined use of pembrolizumab does not necessarily preclude an increased incidence of grade 5 AEs in advanced EC patients. Clinicians should be vigilant regarding the risk of grade 5 AEs in clinical practice. Given the relatively short follow-up duration of the included studies, ongoing monitoring of pembrolizumab safety is essential.

### Strengths and weaknesses

This is the first meta-analysis evaluating pembrolizumab’s efficacy and safety for EC, incorporating RCTs, single-arm, and cohort studies, providing evidence from varying levels. Subgroup analyses explored heterogeneity sources and further corroborated pembrolizumab’s safety and efficacy in treating advanced or recurrent EC. We recognize several limitations, such as most included studies lacked control groups, exhibiting a high risk of bias, while only two were RCTs or cohort studies with small sample sizes. Potential small study effects may have contributed to study heterogeneity and instability.

### Implications for practice and further research

Pembrolizumab has significant efficacy in improving the survival of patients with advanced EC, but the adverse reactions are worthy of attention. Although the incidence of grade 3 and 4 AEs was higher than that of the control group, the safety was generally manageable. Future research will focus on the advantages and disadvantages of pembrolizumab monotherapy or combination therapy and how to control or mitigate adverse reactions and treatment strategies for adverse reactions.

## Conclusion

Pembrolizumab monotherapy or combination significantly improves survival in recurrent or advanced EC and has manageable toxicity albeit with a relatively high incidence of treatment-related AEs.

## Supplementary material

Supplementary Figure 1 RCTs quality assessment chart. (The risk of bias was examined across various domains, including participant selection, intervention performance, outcome detection, participant attrition, reporting, and other potential sources of bias. Each domain was categorized as having a “low risk,” “high risk,” or “unclear risk” of bias. Regarding retrospective studies, NOS scores of 0-4, 5-7, and 8-9 were considered indicative of low, moderate, and high quality, respectively, corresponding to high, moderate, and low risks of bias.)

Supplementary Figure 2 Forest plots of Subgroup outcomes Analysis of **(A)** PFS, **(B)** AE.

Supplementary Figure 3 Funnel plots of **(A)** PFS, **(B)** OS, **(C)** ORR, **(D)** CR, **(E)** AE, **(F)** Grade 3 or 4 AE, **(G)** Grade 5 AE.
