# Supplementary figures and images for: Efficacy and safety of pembrolizumab in patients with advanced endometrial cancer: a systematic review and meta-analysis
Source: Front Oncol. 2025 Feb 4;14:1511301. doi: 10.3389/fonc.2024.1511301 (PMC11832368; doi:10.3389/fonc.2024.1511301)

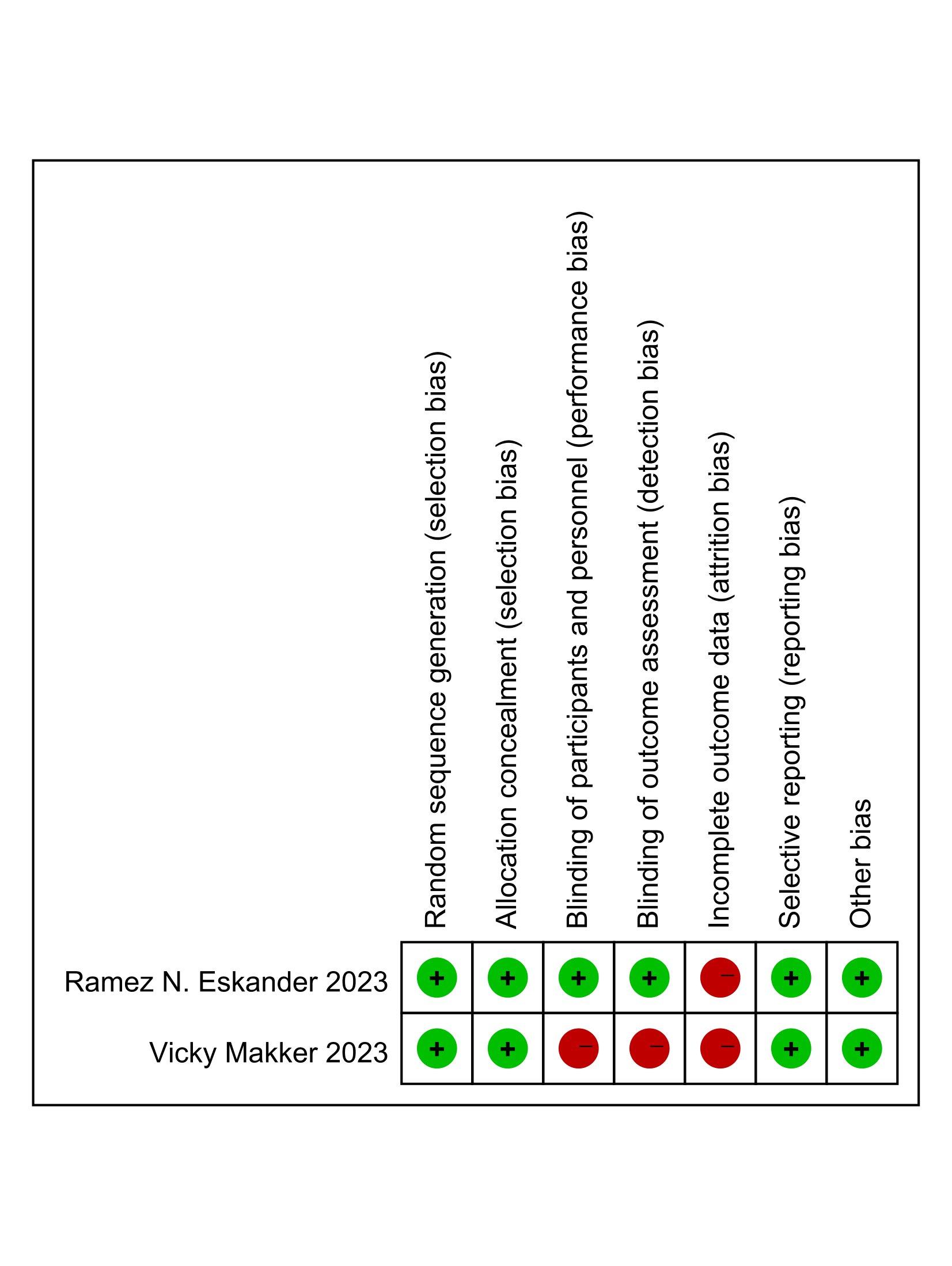

Supplement: Supplementary Figure 1 — RCTs quality assessment chart. (The risk of bias was examined across various domains, including participant selection, intervention performance, outcome detection, participant attrition, reporting, and other potential sources of bias. Each domain was categorized as having a “low risk,” “high risk,” or “unclear risk” of bias. Regarding retrospective studies, NOS scores of 0-4, 5-7, and 8-9 were considered indicative of low, moderate, and high quality, respectively, corresponding to high, moderate, and low risks of bias.) [file Image1.tiff]

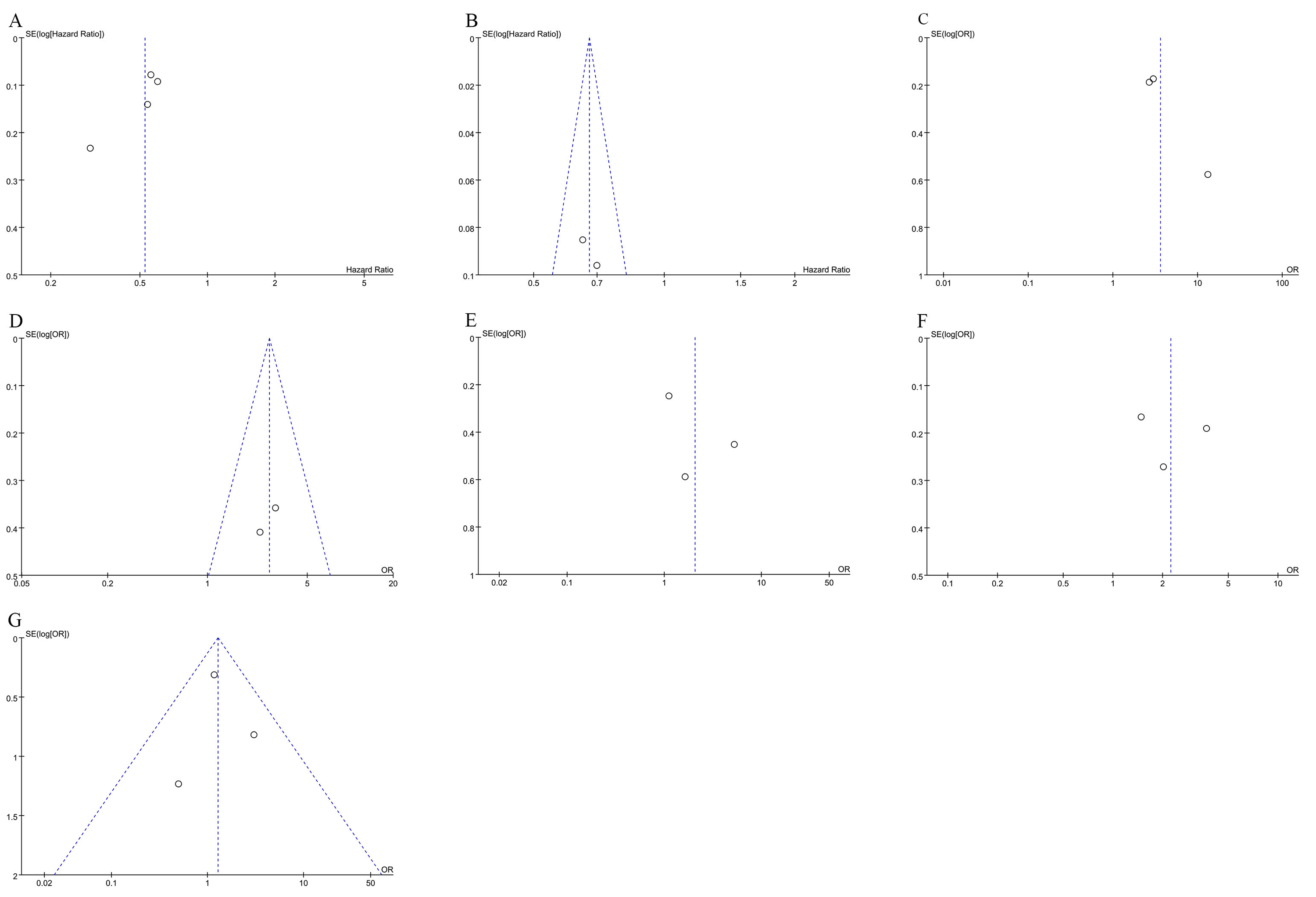

Supplement: Supplementary Figure 2 — Forest plots of Subgroup outcomes Analysis of (A) PFS, (B) AE. [file Image2.tif]

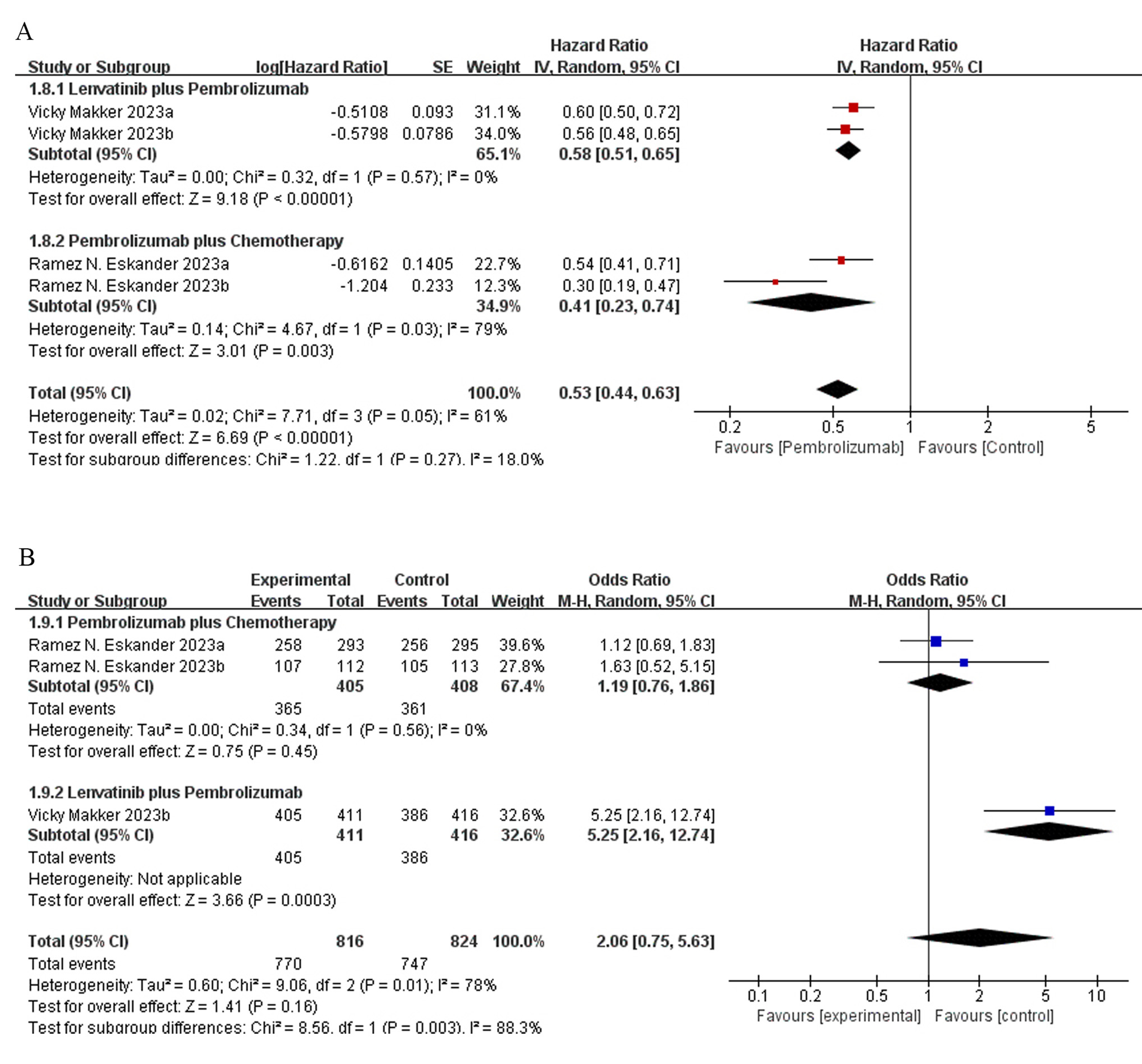

Supplement: Supplementary Figure 3 — Funnel plots of (A) PFS, (B) OS, (C) ORR, (D) CR, (E) AE, (F) Grade 3 or 4 AE, (G) Grade 5 AE. [file Image3.tif]
