# Supplementary material for: Efficacy and safety of pembrolizumab in patients with advanced endometrial cancer: a systematic review and meta-analysis
Source: Front Oncol. 2025 Feb 4;14:1511301. doi: 10.3389/fonc.2024.1511301 (PMC11832368; doi:10.3389/fonc.2024.1511301)
Supplement: Supplementary file 4 [file Table1.docx]

| Supplementary Table 1. Quality evaluation of the eligible studies with Newcastle–Ottawa scale. | | | | | | | | | |
| --- | --- | --- | --- | --- | --- | --- | --- | --- | --- |
| Study | Selection | | | | Comparability | | Outcome | | |
|  | Representative-ness | Selection of  non-exposed | Ascertainment  of exposure | Outcome not present at start | Comparability on most important factors | Comparability on other risk factors | Assessment of outcome | Long enough follow-up (median≥1 year) | Adequacy  (completeness) of follow-up |
| Sneha S. Kelkar  et al. | * | * | * | * | - | - | * | * | * |
| *indicates criterion met; - indicates significant of criterion not met. | | | | | | | | | |
