# Supplementary material for: Efficacy and safety of pembrolizumab in patients with advanced endometrial cancer: a systematic review and meta-analysis
Source: Front Oncol. 2025 Feb 4;14:1511301. doi: 10.3389/fonc.2024.1511301 (PMC11832368; doi:10.3389/fonc.2024.1511301)
Supplement: Supplementary file 5 [file Table2.docx]

| Supplementary Table 2. Quality evaluation of the single-arm studies with JBI scale. | | | | | | | | | | |
| --- | --- | --- | --- | --- | --- | --- | --- | --- | --- | --- |
| Study | (1) | (2) | (3) | (4) | (5) | (6) | (7) | (8) | (9) | (10) |
| David M. O’Malley | Y | Y | Y | Y | Y | N | Y | Y | N | Y |
| Patrick A. Ott | Y | Y | Y | Y | Y | N | Y | Y | N | Y |
| Emma L. Barber | Y | Y | Y | Y | Y | Y | Y | Y | N | Y |
| TStefania Bellone | Y | Y | Y | Y | N | Y | Y | Y | N | Y |
| Vicky Makker | Y | Y | Y | Y | N | Y | Y | Y | N | Y |
| Note: (1) Were there clear criteria for inclusion in the case series? (2) Was the condition measured in a standard, reliable way for all participants included in the case series? (3) Were valid methods used for identification of the condition for all participants included in the case series%? (4) Did the case series have consecutive inclusion of participants? (5) Did the case series have complete inclusion of participants? (6) Was there clear reporting of the demographics of the participants in the study? (7) Was there clear reporting of clinical information of the participants? (8) Were the outcomes or follow up results of cases clearly reported? (9) Was there clear reporting of the presenting site(s)/clinic(s) demographic information? (10) Was statistical analysis appropriate? Y: Yes: N: No. | | | | | | | | | | |
