# Supplementary material for: Efficacy and safety of pembrolizumab in patients with advanced endometrial cancer: a systematic review and meta-analysis
Source: Front Oncol. 2025 Feb 4;14:1511301. doi: 10.3389/fonc.2024.1511301 (PMC11832368; doi:10.3389/fonc.2024.1511301)
Supplement: Supplementary file 6 [file Table3.docx]

| Supplementary Table 3. Baseline characteristics of include studies. | | | | | |  |  |
| --- | --- | --- | --- | --- | --- | --- | --- |
| Authors | Study period/NCT | Country | Study design | Population | Median follow-up(months) | Patients  Experiment/ Control | Sample size  Experiment/ Control |
| Vicky Makker et al.(a) | 2018.6.11-2022.3.1  NCT03517449 | 167 sites in 21 countries | Multicenter, open-label, phase 3 trial RCT | Women（≥18 years of age）with **pMMR**, confirmed advanced, recurrent, or metastatic endometrial cancer of any histologic subtype, except carcinosarcoma and sarcoma | 14.7 | Lenvatinib plus pembrolizumab/ chemotherapy（doxorubicin/ paclitaxel） | 346/351 |
| Vicky Makker et al.(b) | 2018.6.11-2022.3.1  NCT03517449 | 167 sites in 21 countries | Multicenter, open-label, phase 3 trial RCT | Women（≥18 years of age）with **overall** confirmed advanced, recurrent, or metastatic endometrial cancer of any histologic subtype, except carcinosarcoma and sarcoma | 14.7 | Lenvatinib plus pembrolizumab/ chemotherapy（doxorubicin/ paclitaxel） | 411/416 |
| Ramez N. Eskander  et al.(a) | 2019.7-2020.4  /2020.11-2022.12  NCT03914612 | 395 sites in 4countries | Double-blind, randomized, phase 3 trial RCT | Women (≥18 years of age) with **pMMR**, confirmed advanced-stage, metastatic, or recurrent endometrial cancer of any histologic subtype except for carcinosarcoma | 7.9 | Pembrolizumab or placebo along with combination therapy with paclitaxel plus carboplatin | 293/295 |
| Ramez N. Eskander  et al.(b) | 2019.7-2020.4  /2020.11-2022.12  NCT0391461 | 395 sites in 4countries | Double-blind, randomized, phase 3 trial RCT | Women (≥18 years of age) with **dMMR**, confirmed advanced-stage, metastatic, or recurrent endometrial cancer of any histologic subtype except for carcinosarcoma | 12 | Pembrolizumab or placebo along with combination therapy with paclitaxel plus carboplatin | 112/113 |
| Sneha S.Kelkar et al. | 2016.7.1-2018.12.31  - | USA | Cohort study | Women (≥18 years of age) with **MSI-H/dMMR** advanced-stage, metastatic, or recurrent endometrial cancer | - | Pembrolizumab/ chemotherapy | 92/21 |
| David M.O’Malley et al. | 2016.2.1-2020.9.23  NCT 02628067 | 38 sites in 15 countries | An open label, multicohort, phase II , single-arm study | Women (≥18 years of age) with **MSI-H/dMMR**, confirmed advanced-stage, metastatic, or recurrent endometrial cancer | 42.6 | Pembrolizumab | 90 |
| Patrick A. Ott et al. | 2016.2.17  NCT 02054806 | USA | A multicohort, open-label, phase Ib basket trial | Women (≥18 years of age) with **PD-L1 positive**, confirmed advanced-stage, metastatic, or recurrent endometrial cancer | 76.2 weeks | Pembrolizumab | 24 |
| Emma L. Barber  et al. | 2017.9.18-2019.12.12  NCT 02549209 | USA | A single-arm, open-label, multicenter phase II study | Women (≥18 years of age) received up to one prior platinum containing regimen and up to one nonplatinumchemotherapy regimen advanced endometrial cancer | - | Pembrolizumab plus chemotherapy (carboplatin and paclitaxel) | 46 |
| Stefania Bellone  et al. | 2018.4-2020.11.23  NCT 02899793 | USA | An Open-label phase Ib/II Study | Women (≥18 years of age) metastatic, or recurrent advanced endometrial cancer | 25.8 | Pembrolizumab | 24 |
| Vicky Makker et al. | 2015.9.10-2020.8.18  NCT 02501096 | USA | A single-arm, open-label phase II pilot study | Women (≥18 years of age) with measurable, **Lynch-like versus sporadic MSI-H/dMMR** advanced endometrial cancer | 34.7 | Pembrolizumab | 108 |
